# Keyhole Formation by Laser Drilling in Laser Powder Bed Fusion of Ti6Al4V Biomedical Alloy: Mesoscopic Computational Fluid Dynamics Simulation versus Mathematical Modelling Using Empirical Validation

**DOI:** 10.3390/nano11123284

**Published:** 2021-12-03

**Authors:** Asif Ur Rehman, Muhammad Arif Mahmood, Fatih Pitir, Metin Uymaz Salamci, Andrei C. Popescu, Ion N. Mihailescu

**Affiliations:** 1 ERMAKSAN, Bursa 16065, Turkey; faith.pitir@ermaksan.com.tr; 2 Department of Mechanical Engineering, Gazi University, Ankara 06570, Turkey; msalamci@gazi.edu.tr; 3 Additive Manufacturing Technologies Research and Application Center-EKTAM, Gazi University, Ankara 06560, Turkey; 4 National Institute for Laser, Plasma and Radiation Physics (INFLPR), Magurele, 077125 Ilfov, Romania; andrei.popescu@inflpr.ro (A.C.P.); ion.mihailescu@inflpr.ro (I.N.M.)

**Keywords:** laser powder bed fusion, computational fluid dynamics, analytical modelling, shallow and deep keyhole modes, experimental correlation

## Abstract

In the laser powder bed fusion (LPBF) process, the operating conditions are essential in determining laser-induced keyhole regimes based on the thermal distribution. These regimes, classified into shallow and deep keyholes, control the probability and defects formation intensity in the LPBF process. To study and control the keyhole in the LPBF process, mathematical and computational fluid dynamics (CFD) models are presented. For CFD, the volume of fluid method with the discrete element modeling technique was used, while a mathematical model was developed by including the laser beam absorption by the powder bed voids and surface. The dynamic melt pool behavior is explored in detail. Quantitative comparisons are made among experimental, CFD simulation and analytical computing results leading to a good correspondence. In LPBF, the temperature around the laser irradiation zone rises rapidly compared to the surroundings in the powder layer due to the high thermal resistance and the air between the powder particles, resulting in a slow travel of laser transverse heat waves. In LPBF, the keyhole can be classified into shallow and deep keyhole mode, controlled by the energy density. Increasing the energy density, the shallow keyhole mode transforms into the deep keyhole mode. The energy density in a deep keyhole is higher due to the multiple reflections and concentrations of secondary reflected beams within the keyhole, causing the material to vaporize quickly. Due to an elevated temperature distribution in deep keyhole mode, the probability of pores forming is much higher than in a shallow keyhole as the liquid material is close to the vaporization temperature. When the temperature increases rapidly, the material density drops quickly, thus, raising the fluid volume due to the specific heat and fusion latent heat. In return, this lowers the surface tension and affects the melt pool uniformity.

## 1. Introduction

Additive manufacturing (AM) enables customized design, rapid tooling and complicated shape development. It has attracted considerable interest from sophisticated technological applications [1,2], aviation [3,4,5], biomedical research [6,7] and architecture [8,9]. Laser Powder Bed Fusion (LPBF) is a well-known AM technology that offers many advantages, including significantly reduced structural restrictions, high reproducibility and on-time delivery [2,10,11,12,13,14,15].

In the LPBF process, the metallic powder is coated layer-by-layer using a blade or roller, followed by the particles’ fusion via a laser beam on specified locations to produce a three-dimensional (3D) computer-aided design (CAD) model [16]. In LPBF, numerous flaws, including balling, cracking, porosity, or inadequate layer homogeneity, reduce the part quality [15,17,18]. Hence, it is necessary to understand the deformations and the impact of input factors on the melt pool [19,20] dynamics.

Numerous factors, including the laser scanning speed, laser power, particle size distribution (PSD) and layer height, affect the melt pool dynamics and corresponding microstructure, thus, affecting the performance of the produced components [12,21]. However, experimentation is costly and time-consuming to use the hit and try method to optimize the process parameters in the LPBF process. One commonly used approach is to develop simulation models by incorporating all the multi-physical phenomena involved in the LPBF process [22,23]. 

Various systematic attempts have been undertaken to describe the complex melt pool dynamics based on the operating conditions. King et al. [24] investigated the effects of the laser power and scan speed on the surface properties of LPBF parts. It was found that the surface irregularities, deformations and fractures appeared at higher laser scan rates. Gon et al. [25] conducted a study to identify the effect of laser energy density (ED) on the development of the defect.

However, the multi-physical phenomena included in simulating the deposition process was inadequate. Recently, different investigations utilizing a powder-scale mesoscopic framework have been carried out focusing on defect generation. Frazier et al. [26] proposed a simulation method to map the powder interface, including the surface tension, Marangoni tension and recoil. The analyses showed that the powder bed’s thickness could lead to voids and defects in the produced parts.

In the computational fluid dynamics (CFD) process, modelling the process of powder bed formation close to experimental ones is a precondition for a powder-scaled LPBF process. The spreading method primarily includes the deposition of the powder layer with the powder PSD of the given metallic alloys. The Discrete Element Method (DEM) is often used to simulate the metallic powder particle deposition process to achieve this objective.

Panwisawas et al. [27] utilized the DEM coupled with the finite volume method (FVM) to examine the effects of the scanning velocity, power and particle size on the melted region. The analyses showed that the balling defects occurred due to higher laser scanning rates and low laser power. To investigate the formation process of persistent irregularities during the LPBF process, King et al. [24] suggested a comparatively extensive model after considering the recoil pressure and Marangoni effect.

The proposed framework elucidated the powder scattering and pore defects forming process. In addition, the results showed the critical influence of the recoil and Marangoni force on the melt pool dynamics. Yan et al. [28] developed a multi-physical model to define single-track and multiple-track defects formation in the electron beam powder bed fusion (E-PBF) process. They analyzed the effect of energy density and metallic powder bed thickness on the balling effect.

They found that the powder selection and thickness of the metallic powder bed were two critical factors in determining the non-uniformity of its single track. Qian et al. [29] explored the effects of process variables on the manufactured part’s surface quality. They noted that the laser scanning speed is a crucial parameter that is correlated strongly with the molten pool measurements and the surface morphology.

Laser keyhole formation is a fundamental phenomenon in the LPBF process. Interestingly, many efforts have been made to understand the keyhole formation and corresponding porosity development in the LPBF process. Numerous reasons for keyhole pore development have been presented in the literature. For instance, the primary cause of pore development in a keyhole regime is fluctuations and occasional disintegration of the keyhole sidewalls [30].

King et al. [31] explained that the depression regimes might be transformed into keyholes within specific circumstances. They attempted to identify the primary parameters that affect and control the keyhole development in stainless steel. According to Choquet et al. [32], vortex, recirculation and increasing liquid velocities are responsible for pore development and trapping in the keyhole regime. Alternatively, Martin et al. [33] discovered that keyhole pores become locked once the Marangoni effect dominates the buoyancy force, via a combination of in-situ measurement and mathematical modelling.

Cunningham et al. [34] recently recorded the development of a keyhole and pore formation using modern x-ray imaging technology. Even though they saw the occurrences in remarkable spatiotemporal complexity, they did not explain why porosity and keyholes usually originated. In contrary to the purely experimental investigations of King et al. [31] (ex-situ) and Cunningham et al. [34] (operando), Tang et al. [35] investigated keyhole development using a high-fidelity simulation for a metal LPBF process.

Literature analysis identified that laser energy density (ED) controls the thermal distribution at the laser-material interaction regime. If the ED is higher than the material’s melting point, the shallow deep melt pool mode transforms into deep keyhole melt flow mode. The probability of pores and defects formation is much higher in the secondary mode. In this study, analytical and computational fluid dynamics (CFD) models were developed for the laser keyhole formation for LPBF process.

In an analytical model, the density, specific heat and thermal conductivity were calculated for a powder bed by taking into consideration the void ratio defined as the gap between two adjacent particles. Furthermore, the total sample absorption coefficient was calculated based upon the surface absorption coefficient and absorption coefficient by voids. For CFD, the VOF and DEM techniques were implemented. The models were tested and verified in the case of Ti6Al4V material. A close correlation was identified between the experimental and simulation results. In addition, a method was devised to track the melt flow and pattern developed in the shallow deep and deep keyhole melt flow modes.

## 2. Modelling

This section discusses: (a) analytical modelling and (b) computational fluid dynamic (CFD) modelling for laser keyhole formation in the LPBF process.

### 2.1. Analytical Modelling

**Figure 1:**
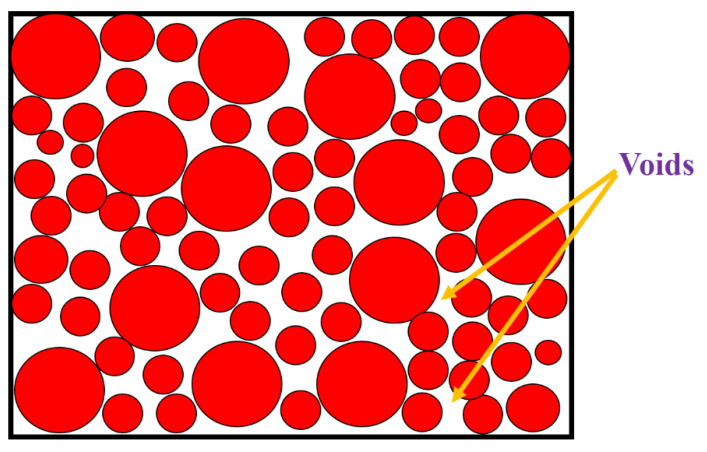
Powder bed schematic with voids.

Figure 1 shows a schematic of the powder bed, a range of perfectly spherical debts, and uniform distribution. The powder particles are uniformly distributed with the void ratio (ε).

The heat conduction equation in the *x*- and *y*-coordinates is expressed as:

(1) $$\frac{\partial^{2}T}{\partial x^{2}}+\frac{\partial^{2}T}{\partial y^{2}}+\frac{1}{k}g\left(x,y,t\right)=\frac{1}{K_{sp}}\frac{\partial T}{\partial t}.$$

Here, *K_sp_* (=*k*/*ρ_sp_C_sp_*) stands for the thermal diffusivity of the powder bed, g(x,y,t) is the volumetric heat source, and *ρ_sp_*, *C_sp_* and $k_{sp}$ are the density, specific heat and thermal conductivity of the powder bed, respectively, that were calculated as:

(2) $$\rho_{sp}=\left(1-\varepsilon \right)\rho_{sb},$$

(3) $$C_{sp}=\left(1-\varepsilon \right)C_{sb},$$

(4) $$k_{sp}=\left(1-\varepsilon \right)k_{sb}$$

where $\rho_{sb}$, $C_{sb}$ and $k_{sb}$ are the solid bulk density, specific heat and thermal conductivity, and ε is the fraction (volume) of voids present in the powder bed. It can be calculated as:

(5) $$\varepsilon =1-\frac{\pi r_{p}^{2}}{S}.$$

(6) $$\begin{array}{ll} S=2(Length\;of & powder\;bed\times Width\;of\;powder\;\;bed)\\ & +2\left(Length\;of\;powder\;bed\times Height\;of\;powder\;bed\right)\\ & +2\left(Width\;of\;powder\;bed\times Height\;of\;powder\;bed\right). \end{array}$$

Here, *r_p_* is the average radius of spherical powder particle, and *S* is the surface area of the powder bed. The thermal distribution ($T_{sp}\left(x,y\right)$) solution of Equation (1) is known in Ref. [36] and was modified by redefining the total sample absorption coefficient ($\alpha_{sp}\left(z\right)$) based upon the surface absorption coefficient (*r_sp_*) and absorption coefficient voids ($\alpha_{sp}$) [37]:

(7) $$T_{sp}\left(x,y\right)=T_{\infty }+\frac{P\alpha_{sp}\left(z\right)}{4\pi Rk}exp\left(\frac{V_{x}\;\left(R+x\right)}{2K_{sp}}\right).$$

(8) $$\alpha_{sp}\left(z\right)=r_{sp}\delta \left(z\right)+\alpha_{sp}.$$

Here, *P* is the laser power, $T_{\infty }$ is the room temperature, *V_x_* is the laser scanning speed, and *R* is the laser beam-powder bed distance, expressed as:

(9) $$R=\sqrt{x^{2}+y^{2}}.$$

The correlation among the laser power, laser intensity (*I*), and laser spot (*r_l_*) is expressed as:

(10) $$P=\frac{I\pi r_{l}^{2}}{2}.$$

After substituting the above expression in Equation (7), one obtains:

(11) $$T_{sp}\left(x,y\right)=T_{\infty }+\frac{Ir_{l}^{2}\alpha_{sp}\left(z\right)}{8Rk}exp\left(\frac{V_{x}\;\left(R+x\right)}{2K_{sp}}\right).$$

In Equation (8), δ(z) is the Dirac-delta function as a function of substrate depth (*z*). We next consider a powder layer with several voids, as shown in Figure 1. When a laser beam passes through a powder layer containing voids with a width (*w_v_*) and depth (*d_v_*), the incident laser beam will experience numerous reflections before coming out of the void. It, in return, increases the bulk laser absorption coefficient ($\alpha_{sp}\left(z\right)$). In Equation (3), *r_sp_* is a dimensionless value; therefore, letting *r_sp_* equal to *w_v_*/*d_v_* gives:

(12) $$\alpha_{sp}\left(z\right)=\left(w_{v}/d_{v}\right)\delta \left(z\right)+\alpha_{sp}.$$

Here, $w_{v}$ and $d_{v}$ are the width and depth of a void, respectively.

### 2.2. Numerical Modelling

The powder development and deposition process computation can be separated into two stages: (a) initially, a range of particles falls directly on the surface to generate a powder stack, and (b) subsequently, the powder re-coater spreads a layer of powder particles uniformly, thus, generating a powder layer at the building platform. An interaction method with the non-linear Hertz–Mindlin elastic equation is used to measure the elastic actual contact force [38], while the damping factor is theoretically applied to acknowledge the dissipation of mechanical energy [39,40,41].

The natural contact force and damping force in elastic materials, at the overlap between such interacting particles, is always perpendicular to the plane. No micro-slip approach is introduced in the tangential route to accommodate for elastic contact force [38]. The particle size distribution (PSD) for Ti6Al4V, provided by ERMAKSAN, Turkey, is D10 = 19 µm, D50 = 30 µm and D90 = 46 µm. The discrete element modelling (DEM) module from Flow Science, USA was used to model the powder layer deposition for Ti6Al4V metallic powder particles throughout this research.

**Figure 2:**
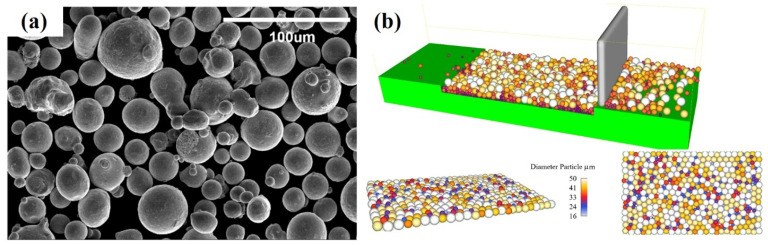
(**a**) Scanning electron microscopy images of Ti6Al4V powder particles and (**b**) simulated powder bed using discrete element modelling.

The morphology of the Ti6Al4V powder particles, measured using scanning electron microscopy (SEM), along with the simulated powder bed using PSD, are shown in Figure 2. The packing density of the powder particles is 65%, and the temperature-dependent physical properties were utilized for Ti6Al4V powder particle CFD modelling.

The FLOW-3D 11.2v CFD software and additive manufacturing from Flow Science, Santa Fe, NM, USA, were used to develop and integrate a CFD framework. In this research, multiple variables and generalizations have been made: (a) the melting is assumed incompressible Newtonian throughout the melt stream, and (b) the change in mass owing to metal evaporation is taken into account. Continuity of mass, momentum and energy conservation are all solved by using the following equations:

(13) $$\nabla \cdot \vec{v}=0.$$

(14) $$\frac{\partial \vec{v}}{\partial t}+\left(\vec{\nu }\cdot \nabla \right)\vec{v}=-\frac{1}{\rho }\nabla \vec{P}+\mu \nabla^{2}\vec{v}+\vec{g}\left[1-\alpha \left(T-T_{m}\right)\right]g\left[1-\alpha \left(T-T_{m}\right)\right].$$

(15) $$\frac{\partial h}{\partial t}+\left(\vec{v}\cdot \nabla \right)h=\frac{1}{\rho }\left(\nabla \cdot k\nabla T\right).$$

where *v* defines the velocity profile, $\vec{P}$ identifies pressure, *μ* specifies viscosity and $\vec{g}$ represents the gravity function, α specifies the coefficient of thermal expansion, ρ specifies density, *h* denotes specific enthalpy and *k* is heat conductivity. A volume of fluid (VOF) model has been applied as shown in Equation (18) [42]:

(16) $$\frac{\partial V_{F}}{\partial t}+\nabla \left(\vec{\nu }\cdot V_{F}\right)=0.$$

The metal volume fraction ($V_{F}$) is used to specify the fluid: cells are said to be completely fluid if, $V_{F}=1$, whereas if the cells that have no fluid inside them will have $V_{F}=0$. Melt pool dynamics usually vary due to thermo-physical characteristics, vapor suppression and penetration. Since the Rosenthal method is re-derived from the heat equation and eliminates evaporation, convection and the Marangoni effect [43,44], the equivalent term in Equation (17) shows the melt pool diameter extracted from the Rosenthal formula [45]. It explains the significance of thermo-physical features in melt pool heterogeneity during heat transfer [43] as:

(17) $$\omega =\sqrt{\frac{8}{\pi e}\cdot \frac{P\eta }{\rho C_{p}V\left(T_{m}-T_{0}\right)}.}$$

Here, the melt pool width is specified by ω, the beam power is specified by *P*, laser beam absorptivity is *η*, density is  ρ and $C_{p}$ is the heat capacity. Furthermore, *V* specifies the laser beam scanning speed and the melting temperature is specified by $T_{m}$ and the pre-heating level is specified by $T_{0}$. Thermal independence and thermophysical conductivity to measure the melt pool size are presumptions in determining the Rosenthal solution. The impact of recoil pressure and vapor suppression on melt pool size is also considered [46]. Equation (18) is used to determine the recoil pressure:

(18) $$P_{S}=A\cdot exp\left\{B\left(1-\frac{T_{V}}{T}\right)\right\}.$$

The secondary coefficient *A* is equal to $\beta P_{0}$, *β* ∈ [0.54, 0.56], and $P_{0}$ is the atmospheric pressure. The *B* = $\Delta H_{V}/RT_{V}$, where $\Delta H_{V}$ is an accumulated vaporization heat [46], *R* stands for gas constant and $T_{V}$ is the saturation temperature [41,46,47,48]. Here, the laser energy density is distributed in accordance with a Gaussian curve. The laser beam scanning speed is constant and the energy density (*q*) of the beam is expressed as [46]:

(19) $$q=\frac{2Ap}{\pi R_{b}^{2}}exp\left[-2\frac{{\left(x-\nu t-x_{0}\right)}^{2}+{\left(y-y_{0}\right)}^{2}}{R_{b}^{2}}\right],$$

where *A* denotes the particle bed’s beam absorbance, *p* denotes the laser power, *R_b_* denotes the laser beam spot radius, *v* denotes the scanning rate, and $x_{0}$ and $\;y_{0}$ represent the original position of the laser beam center [46]. The beam radius, $R_{b}$, is set as 27.5 m. However, evaporation is critical when considering the hot surface of the melt pool due to convection and radiation. As a consequence, the governing equation [46] may be represented primarily on the melt pool surface as:

(20) $$\frac{\partial T}{\partial \vec{n}}=q-h_{C}\left(T^{1}-T_{0}^{1}\right)-\sigma_{0}\in \left(T^{4}-T_{0}^{4}\right)-q_{evap}.$$

Here, $h_{c}$ is the coefficient of convection heat transfer, $T_{0}$ is the room temperature, $\sigma_{0}$ is the Stefan-Boltzmann constant and ∈ is an emissivity measurement. The temperature distribution owing to evaporation $\left(q_{evap}\right)$ is represented as:

(21) $$q_{evap}=\omega_{0}L_{v}=exp\left(2.52+6.121-18836T-0.5logT\right)L_{v}.$$

where $\omega_{0}$ is the evaporation rate. To calculate the mass flow rate, following equation has been applied:

(22) $$\dot{m}=\int \rho \cdot \vec{v}\;d\vec{A}.$$

Here, $\vec{v}$ is velocity and ρ is density. Simulation values were assumed for thermal distribution, top and bottom width of the keyhole to reflect a close correlation with experimental results and does not contain real measurements in the simulations.

## 3. Materials and Methods

To validate the simulation results, experimental analyses from the study of Gong et al. [49] were utilized in the case of LPBF of Ti6Al4V. In this study, single tracks equivalent to 1600 µm (length) were deposited. Following on, the authors performed the metallographic experiments to determine the keyhole dimensions. Table 1 collects the thermo-physical properties of Ti6Al4V used to conduct the simulations.

The length, width and depth of the substrate are equal to 2500 µm, 200 µm and 400 µm, respectively, and the thickness of the powder layer is 70 µm. Two types of operating conditions were used: (a) for shallow depth keyhole, the laser power is equal to 195 W and laser scanning speed is 1000 mm/s, and (b) for deep keyhole, the laser power is 195 W and laser scanning speed is 400 mm/s. For simulations, adiabatic bottom and slip-wall sides are used in the model.

If necessary, the material can eject itself from the x-z plane, which is a symmetry barrier with constant pressure and temperature (ambient temperature). There are 6,288,368 cells with an average size of 3.3 µm and an aspect ratio very near to one, which is suitable for surface tracking algorithms such as volume of fluid. For computations, a discrete element calculation compiled in the FLOW-3D DEM module was used to determine the distribution of powder particle during the powder laying setup. Table 2 collects the parameters used to carry out analytical and CFD simulations.

## 4. Results and Discussions

**Figure 3:**
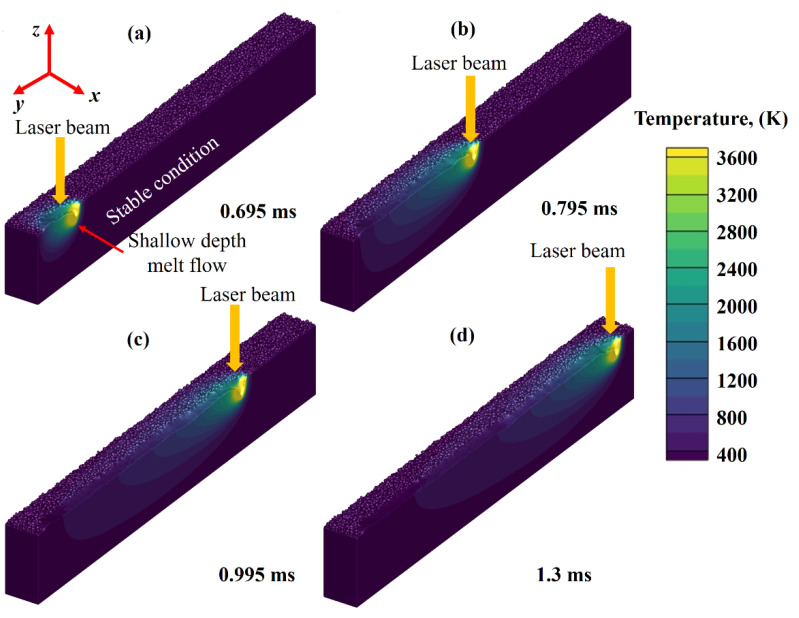
Temperature field contour formation at various time intervals (**a**) 0.695 ms, (**b**) 0.795 ms, (**c**) 0.995 ms and (**d**) 1.3 ms.

Figure 3a–d depict the temperature contour and melt pool boundary at four different times in the SLM process. Figure 3a shows that the temperature immediately below the laser irradiation point rises to almost 3600 K, but the surrounding powder layer is still at 400 K temperature. The thermal resistance is considerable due to the air between the powder particles and the comparatively low particle–particle contact areas, thus reducing the speed of laser transverse heat waves.

This implies that the bulk material conducts heat at a considerably higher rate than the powder bed. There are two kinds of melt pool patterns often seen in laser additive manufacturing processes: (a) shallow mode and (b) deep keyhole mode [51,52]. In the first category, the material is heated from the top down, and the laser energy used exceeds the rate at which heat is radiated, resulting in a melt pool forming. For the second category, highly concentrated laser energy is focused on a given area, resulting in a material temperature higher than the boiling point.

In depression mode, the heat source melts both the exterior and interior surfaces of the material. From simulations, the formation of a shallow mode melt pool below the laser can be identified clearly. The recoil pressure and Marangoni-induced flow are responsible for this shallow melt pool formation; however, the recoil pressure has the highest impact among all the previously described phenomena [53]. When the melt pool has sufficiently penetrated downwards, it will continue its way to the back of the melt, owing to the liquid’s high deformability.

At this instant, the laser beam rays are mostly either unable to enter that far or have lost most of their energy due to a considerable number of collisions, resulting in keyhole’s local temperature declination at the tail. This low-temperature zone will cause a local rise in surface tension and a significant decrease in recoil pressure that will initiate the formation of the pores.

**Figure 4:**
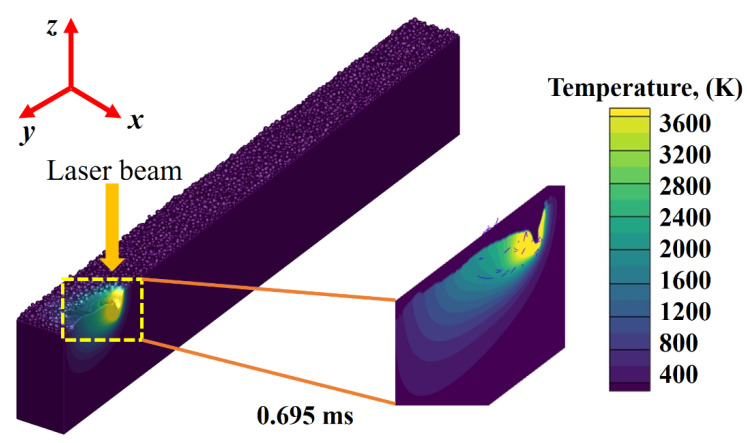
Detailed view of shallow depth melt mode with temperature field at 0.695 ms.

For better illustration and visualization, a detailed view of the shallow mode melt flow pattern is provided in Figure 4, along with the thermal distribution contours formation.

**Figure 5:**
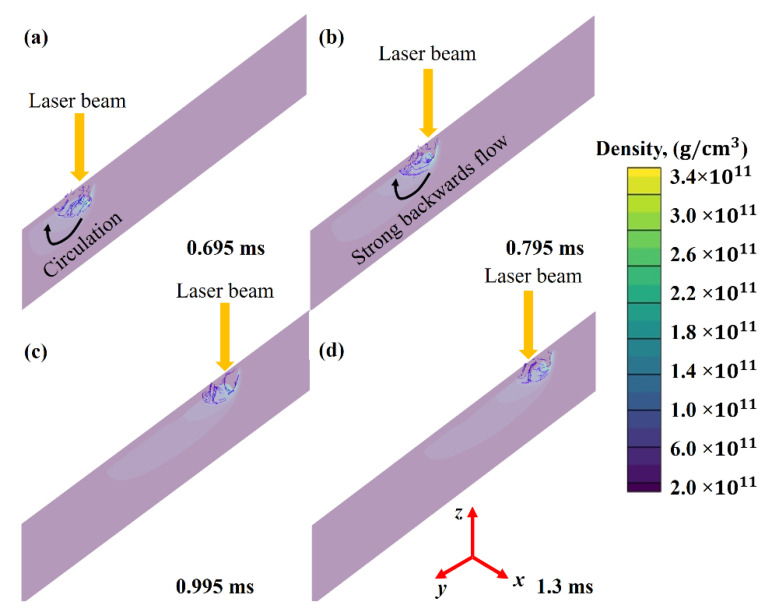
Melt flow stream traces formation at various time intervals (**a**) 0.695 ms, (**b**) 0.795 ms, (**c**) 0.995 ms and (**d**) 1.3 ms.

Figure 5a–d show the stream traces in the developed keyhole at four different time intervals. Despite the presence of the Marangoni effect, the downward movement is primarily controlled by the presence of a high recoil pressure acting on the top surface. Figure 5 shows a tidy clockwise circulation at the rear side of the melt pool, which is characteristic of the LPBF process [27,28] or welding processes [38,44,45].

Keyhole development and subsequent expansion are usually caused by both a strong downward flow and a hotspot. When a hotspot forms below a depression zone owing to laser beam collisions, a strong downward flow will carry all the heat stored at this point, together with all streamlines inside the melt pool. It will result in a deeper depression zone inside the melt pool since the recoil pressure grows exponentially as the temperature increases [25]. According to Martin et al. [31], the reflections on the front of the depressions will cause vaporization on its rear. Figure 5a–d provide the visual perception of the shallow-depth melt pool.

**Figure 6:**
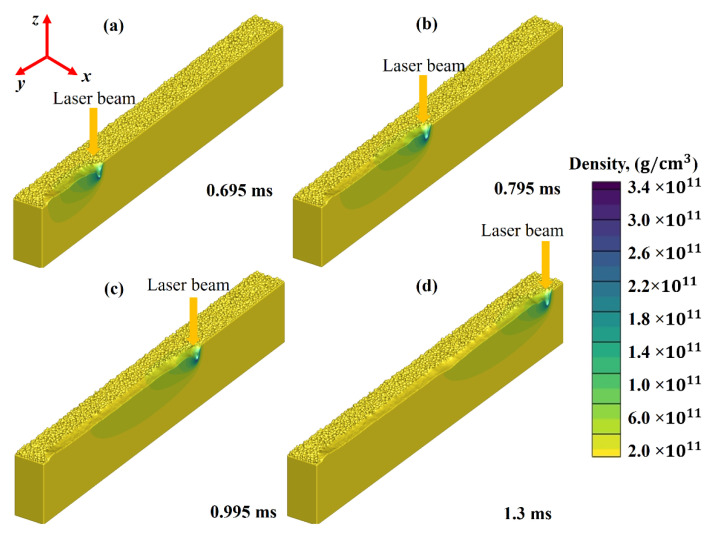
Density evolution of the melt pool at various time intervals (**a**) 0.695 ms, (**b**) 0.795 ms, (**c**) 0.995 ms and (**d**) 1.3 ms.

Figure 6a–d show the melt-pool density evolution generated at 0.695 ms, 0.795 ms, 0.995 ms, and 1.3 ms, respectively. The results show that, with rising temperature, material density falls rapidly due to its specific heat and latent heat of fusion. It, in turn, increases the fluid volume. It is essential to point out that the volume rises dramatically when density drops, lowering the surface tension (ST). Thermocapillary convection, or Benard–Marangoni convection, is another name for this kind of ST. The ST difference mostly defines the melt pool.

When the ST difference between the two ends of a liquid develops, a strong pull is produced from the high ST end to the low ST end. The figures show that when the powder layer is irradiated and the laser beam moves away, the heat begins to dissipate from the irradiated regime, thus solidifying the melted region. It is worthy of mentioning that density changes are inversely proportional to temperature variations. Five main driving factors have been identified to melt flow, including Marangoni flow, vaporization and high-speed clouds of vapour, hydraulic pressure and buoyancy [54,55,56,57].

“Benard–Marangoni convection” occurs when a material has a negative thermal surface tension coefficient and moves from a high to a low thermal regime. “Recoil pressure” is perpendicular to the evaporated surface because of the internal compression produced by “vaporization”. Shear force may be generated in a “high-speed vapor cloud” via friction at the gas-liquid periphery. The capacity to transmit energy via hydrostatic or hydrodynamic pressure is referred to as “hydraulic pressure”. The “buoyancy force” compels the molten material to follow the density gradient. In LPBF, splashing and spattering will occur if any of the forces mentioned above exceed the threshold value.

**Figure 7:**
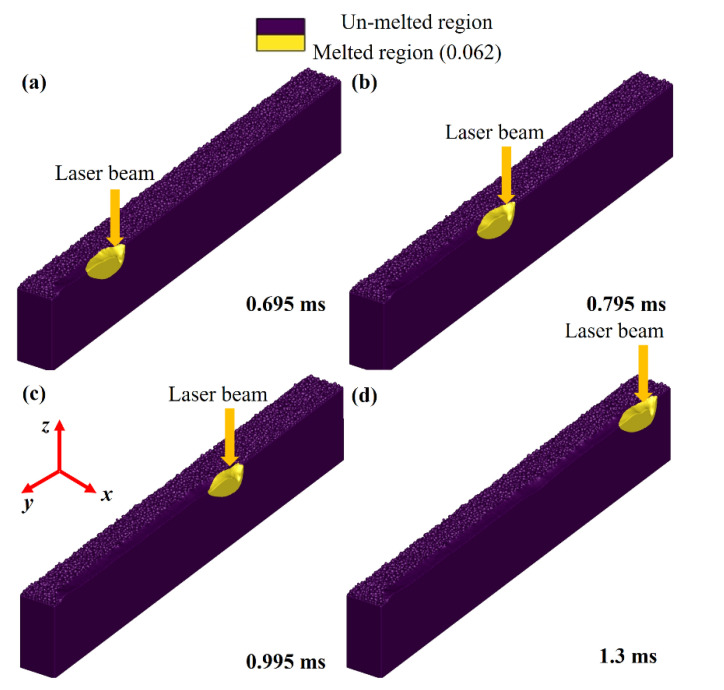
Un-melted and melted regions at different time intervals (**a**) 0.695 ms, (**b**) 0.795 ms, (**c**) 0.995 ms and (**d**) 1.3 ms.

Figure 7a–d show a differentiation between the un-melted and melted regions attained in LPBF of Ti6Al4V. The yellow area identifies the fraction of melted regime during the printing process, while the purple colour displays the un-melted area. The results identify that as the laser beam moves away from the irradiated region, the liquified area experiences conduction, convection, and radiation that causes solidification of the melt pool, resulting in layer formation.

**Figure 8:**
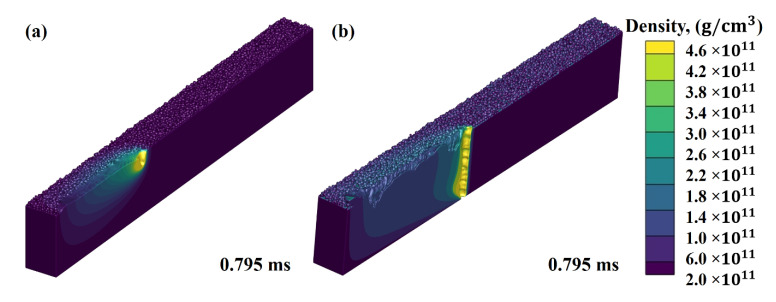
Transformation from shallow depth melt flow to deep keyhole formation when laser power increased from (**a**) 170 W to (**b**) 200 W.

Figure 8a,b display the effect of laser power on the transformation of shallow mode melt flow (SMMF) to deep keyhole melt flow (DKMF). A direct correlation can be identified between laser power and the depth of keyhole formation from the results. It can be explained by the fact that the powder layer in the compact form has several voids, defined as the average distance between two adjacent powder particles. When the laser power increases, the energy transferred to a given area also increases and optical rays experience multiple reflections within the vids. It, in return, elevates the laser beam absorption coefficient. Hence, these reflections will transform the SMMF to DKMF. This effect is often described as “laser light capture” by voids [33].

**Figure 9:**
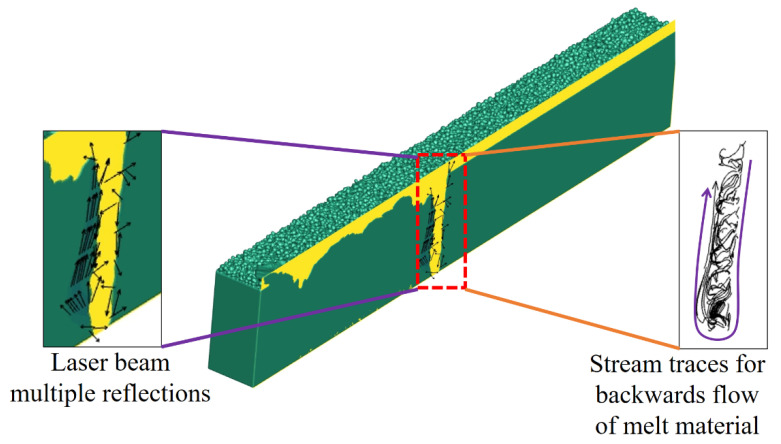
Stream traces and laser beam multiple reflections in deep keyhole melt flow mode.

As can be seen in Figure 9, the DKMF generates numerous laser beam reflections and stream traces. The laser energy density is much higher in DKMM, thus pushing the material towards a vaporization regime. The porosity occurs if the solid front hits quickly before their escape. Due to a higher temperature distribution in DKMM, the probability of pores forming is much higher than in SMMF, as the liquid material is close to the vaporization zone. In addition, the melt flow, in both DKMM and SMMF modes, exhibited a clockwise direction.

**Figure 10:**
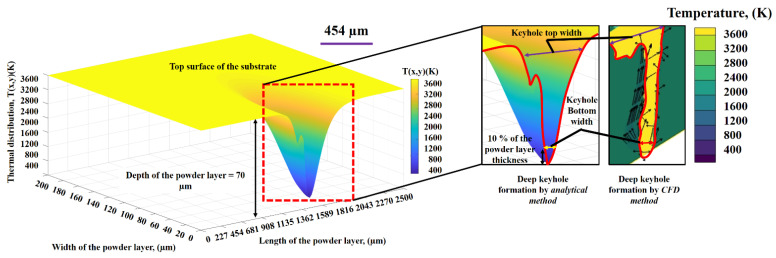
A comparison between analytical and CFD simulation results for peak thermal distribution value in the deep keyhole formation.

Figure 10 shows a comparison of thermal distribution in the laser keyhole formation estimated by analytical and CFD methods. It is worthy to mention that only peak thermal value has been adopted for comparison. For the analytical simulations, a MATLAB software with user-defined codes was applied. A powder layer having length = 2500 µm, width = 200 µm and depth = 70 µm were selected. The model estimated the keyhole dimensions and the thermal distribution inside the induced laser keyhole. From the results, it can be analyzed that the analytical model was able to estimate results close to the CFD results in terms of deep keyhole profile and dimensions.

The top width of the keyhole was measured just below the laser-powder layer interaction regime, while, the keyhole’s bottom width was calculated at a distance equal to 10% of the powder layer thickness to avoid blind hole having zero width. This blind hole is resulted due to the Gaussian heating source utilization in the case of analytical modelling that shows dominant effects. However, for CFD modelling, this effect lessens due to the inclusion of VOF and DEM techniques. To keep the homogeneity in the keyhole dimension results, the same methodology, as in analytical modelling, was applied for CFD results.

**Figure 11:**
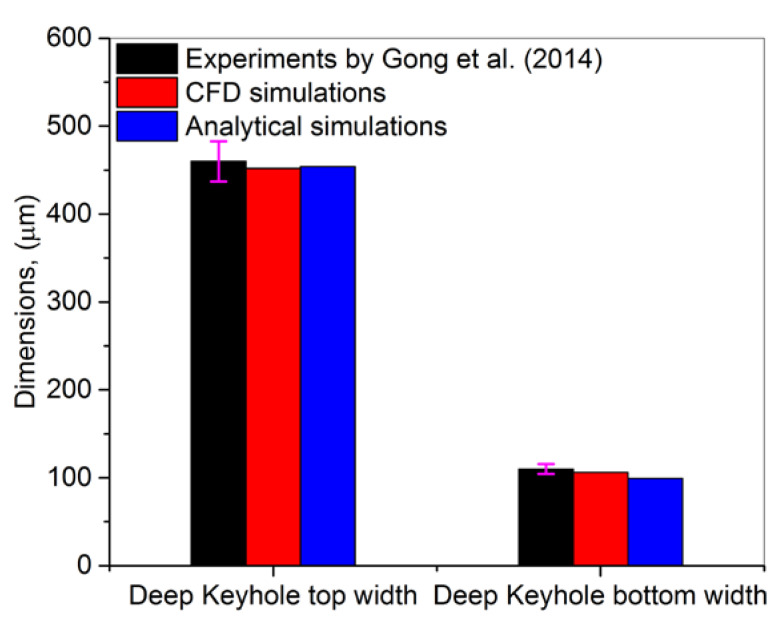
A comparison among experiments [49], CFD and analytical simulations for deep keyhole top width and bottom width.

Figure 11 shows a comparison among experiments, CFD and analytical simulations. It can be seen that the CFD model presented results close to the experimental results with a deviation of 2%. However, these deviations increase by 5% while implementing the analytical model. It is because the analytical does not consider the volume of fluid and discrete element modelling technique, which is available in the CFD simulations.

**Figure 12:**
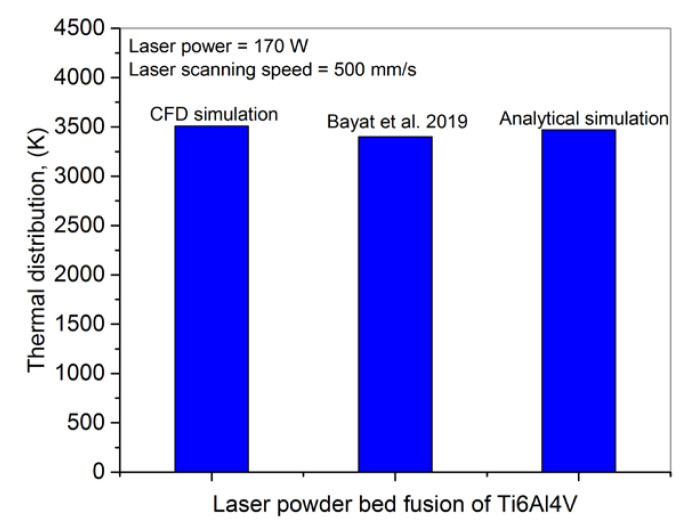
A comparison for thermal distribution among CFD simulation, literature result [58] and analytical computation for keyhole formation.

Figure 12 presents a comparison among current CFD model, Bayat et al. [58] simulation results and present analytical computations. It can be seen that the current CFD and analytical models presented results near to the Bayat et al. [58] results except a few deviations.

## 5. Conclusions

In this paper, we studied shallow and deep laser keyhole formations using mathematical and computational fluid dynamic (CFD) modelling. For CFD, the volume of fluid and discrete element modeling techniques were applied, whereas an analytical model based on mathematical equations was presented by including the laser beam absorption by the powder bed voids and surface. The melt pool dynamics and flow behavior were discussed in detail. Experimental, CFD simulation and analytical, computational results were compared quantitatively. The CFD and analytical computation results correlated with the experimental analyses with deviations of 2% and 5%, respectively. The following findings were made based on the current research:

- The temperature below the laser irradiation point rises rapidly in LPBF, whereas the powder layer around it remains at a localized temperature. Due to air between the powder particles and the relatively low particle–particle contact areas, laser transverse heat waves have a slower speed because of the high thermal resistance.
- Two types of laser keyholes can be generated during the LPBF process: shallow and deep keyholes. The mode type can be controlled and defined to an extent by the energy density. Increasing the energy density leads to an increase in the amount of energy delivered to a given region, and optical rays encounter numerous reflections due to voids available in the deposited powder layer. In return, this increases the laser beam absorption coefficient. As a result, the shallow keyhole converts into a deep keyhole.
- The deep keyhole usually generates numerous laser beam reflections and stream traces. A deep keyhole experiences a larger energy density compared with a shallow keyhole, pushing the material towards vaporization. The porosity usually occurs if the solid front hits quickly before they escape from the melt pool. Due to an elevated temperature distribution in the deep keyhole, the probability of pores forming is much higher than in a shallow keyhole, as the liquid material is close to the vaporization zone. However, both shallow and deep keyholes exhibited a clockwise direction.
- Due to the specific heat and fusion latent heat, the results demonstrated that, as the temperature increases, the material density decreases rapidly, elevating the fluid volume. This, in return, reduces the surface tension (ST), and is also known as Benard–Marangoni convection or thermocapillary convection. The ST difference largely determines the size of the melt pool. When a liquid’s ST difference widens, a significant pull arises from the high ST end toward the low ST end.

This study provides a cost- and time-effect modelling technique to identify and control shallow and deep keyhole formation based on the operating conditions.

## Figures and Tables

**Table 1:** The thermo-physical properties of Ti6Al4V [49].

Parameters	Values (Units)
Solidus temperature	1878 (K)
Liquidus temperature	1928 (K)
Boiling temperature	3533 (K)
Latent heat of fusion	286 (kJ/Kg)
Latent heat of evaporation	9830 (kJ/Kg)
Viscosity	0.005 (Kg/ms
Surface tension	1.68 (N/m)
Surface tension gradient	−0.00026 (N/mK)

**Table 2:** Parameters for analytical and CFD simulations [50].

Parameter	Value	Parameter	Value
Substrate length × width × depth	2500 µm × 200 µm × 400 µm	Average size of cell for CFD simulations	3.3 µm
Powder layer thickness	70 µm	Ti6Al4V particle size distribution	D10 = 19 µm, D50 = 30 µm and D90 = 46 µm
Laser power	195 W	Laser beam entire absorption coefficient	0.25
Laser scanning speed	400 mm/s, 1000 mm/s	Number of cells generated for CFD simulation	6,288,368
Effective laser beam radius where the heat flux is 1/e^2^ of its maximum value	25 µm		

## Data Availability

Not applicable.
